# 

**Published:** 1917-08-25

**Authors:** 


### Scottish Women's Hospitals.

### MORE PAY FOR FULLY TRAINED NURSES AND OTHERS.

The Committee of the Scottish Women's Hospitals, which have at the present date six units at work with the armies of the Allies, have decided to depart from their original procedure, whereby no distinction in pay was made between nurses of different grades of training, and have instituted a difference in the remuneration of "fully trained" and "not fully trained" nurses. Fully trained nurses receive salary at the rate of £50 per annum, and have the title of Sister. Nurses with less than three years' training receive remuneration at the rate of £45. In both cases there is a rise of salary at the rate of £5 per annum after one year's service. Nurses of both classes receive uniform and travelling expenses.

Gifts to the Hospital.

### Gifts and Bequests.

Lady Swaythling has presented hospital requisites, to the value of £400, for the use of the Rumanian Red Cross.

Sir W. J. Thomas has endowed a bed at the King Edward VII.'s Hospital, Cardiff, at a cost of 1,000 guineas, in honour of Lady Thomas.

Mr. William Taylor, of Perth, left one-tenth of the residue of his estate each to the Dunfermline and West Fife Hospital and the Royal Infirmary, Stirling.

Mr. Albert Ashton, of Sheffield, bequeathed £200 each to the Sheffield Royal Hospital and the Sheffield Royal Infirmary, and numerous smaller sums to other institutions.

Ancoats Hospital has received a legacy of £100 from the executors of the late Mr. John Banks, and £500 from the executors of the late Miss Mary Barnes, of Wilmslow.

Mr. Robert Colver, J.P., left £300 each to the Sheffield Royal Hospital and Royal Infirmary, and £200 each to the Jessop Hospital for Women and the Free Hospital for Children, Sheffield.

Miss Elizabeth Durrant, of Bournemouth, left £500 to the Royal Victoria Hospital, Bournemouth, £300 to the Norfolk and Norwich Hospital, £250 to the London Hospital, and £500 to the Hospital for Incurables at Putney.

The late Mr. C. W. Ware, of Ripon, made a number of charitable bequests, amongst them being £4,000 to the British Home and Hospital for Incurables, Streatham, and £2,000 to the Home for Destitute Crippled Children at Gosport. The ultimate residue of Mr. Ware's estate was left to the Royal Victoria Infirmary, Newcastle-on-Tyne.

H.M. The King has been graciously pleased to contribute £200 towards King George's Fund for Sailors.

Mr. Charles Morgan, of Liverpool, bequeathed £100 to the Liverpool Skin Hospital.

Mr. Septimus William Richard Moate, of Overstone, Cutcliffe Grove, Bedford, whose estate has been sworn at £8,290, left the residue to the West Hertfordshire Infirmary.

A cot in the children's ward of the Epping Cottage Hospital is to be endowed as a memorial to the famous cricketer, the late Mr. C. E. Green. Over £500 has already been subscribed.

The honorary secretaries of King Edward's Hospital Fund for London have received at the Bank of England the sum of £35,000, being a contribution from a donor who desires his gift to be so acknowledged.

Mr. W. S. Handley and the Hon. H. B. Portman have given £100 each, and Messrs. S. B. and J. B. Joel £210, towards the Research Fund in connection with the Bland-Sutton Institute of Pathology attached to Middlesex Hospital.

Sir Henry Arthur Wiggin, Chairman of the North Staffordshire Railway Company, bequeathed £100 each to the Birmingham and Midland General Hospital for Children, the Birmingham General Hospital, and the Staffordshire General Infirmary.

The East Lancashire Branch of the British Red Cross Society has received for the provision of homes for disabled sailors and soldiers, 600 guineas, the result of a sale of work organised by the lady superintendent of the Manchester Royal Infirmary. Many of the articles sold were made by the nursing staff and domestic servants of the Royal Infirmary.

### Matrons' and Sisters' Appointments.

GLOUCESTERSHIRE ROYAL INFIRMARY.—Miss Florence Tillson has been appointed matron. She was trained at the London Hospital, and has been out-patients' and home sister at the Hospital for Women, Shaw Street, Liverpool; assistant-matron at the Ophthalmic Schools, Brentwood; and house-keeping sister at the General Hospital, Swansea, where she has also done assistant-matron's duties.

INFECTIOUS DISEASES HOSPITAL, ABRAM, NEAR WIGAN.—Mrs. L. Scott has been appointed matron. She was trained at the Royal Victoria Infirmary, Newcastle, and has held the post of matron at Harrogate Fever Hospital and at Gravesend Borough Sanatorium.

RAYMEAD CHILDREN'S HOSPITAL, MAIDENHEAD.—Miss K. Josephine Speirs has been appointed matron. She was trained at the Northern Hospital, Manchester, and has since been staff nurse on the private nursing staff of the Children's Hospital, Great Ormond Street; ward, superintendent, and home sister at Queen Mary's Hospital for Children, Carshalton; and matron of Westmorland Sanatorium and of Bleakdown Auxiliary Military Hospital, West Byfleet. Miss Speirs holds the certificate of the Royal Sanitary Institute, and has held the position of lecturer on hygiene at Darlington Training College.

BARTHOLOMEW HOSPITAL, GOOLE.—Miss Edith Brown has been appointed nurse-matron. She was trained at the General Infirmary, Burton-on-Trent.

CHARING CROSS HOSPITAL.—Miss Ethel Maude Eaves has been appointed sister midwife, and Miss M. V. Helmore Wood casualty sister. Miss Eaves was trained at Preston Infirmary, and has since been sister at the Clayton Hospital, Wakefield, theatre sister at the East Suffolk Hospital, Ipswich, nurse in charge of Saint Marylebone General Dispensary, and matron of the Duchess of Marlborough's Maternity Hospital. Miss Wood was trained at Charing Cross Hospital, and has since been working at the Victoria Hospital for Children at Chelsea.

CROESNEWYDD AUXILIARY MILITARY HOSPITAL, WREXHAM.—Miss E. M. Richards has been appointed ward sister. She was trained at Borough Fever Hospital, Bolton, and the Royal Albert Edward Infirmary, Wigan.

DEACONESS HOSPITAL, EDINBURGH.—Miss Edith B. Gammon has been appointed sister. She was trained at York County Hospital and at York Maternity Hospital, at each of which institutions she has acted as sister.

HACKNEY UNION INFIRMARY, 230 HIGH STREET, HOMERTON.—Miss Beatrice Mary Barford has been appointed superintendent night nurse, and Miss Edith Jones surgical ward and theatre sister. Miss Barford was trained at Hackney Union Infirmary, where she was successively staff nurse and sister. Miss Jones was trained at Edmonton Union Infirmary.

PRINCESS ALICE MEMORIAL HOSPITAL, EASTBOURNE.—Miss Ethel Wakeman and Miss Nellie Pinder have been appointed sisters. The former was trained at Hereford General Hospital, where she was later night sister; and the latter at the Royal Berkshire Hospital, Reading.

ROCHDALE INFIRMARY.—Mrs. Emily Low has been appointed sister. She was trained at the Leeds General Infirmary and under the Metropolitan Asylums Board. She has held a position on the staff of the 2nd Western General Hospital, Manchester, and was sister at the Royal Albert Edward Infirmary, Wigan.

ROYAL INFIRMARY, PERTH.—Miss Jean S. Jeffries has been appointed sister. She was trained at the Coventry and Warwickshire Hospital, and has since done private nursing and held the post of sister at the Women's Hospital, Glasgow.

**NOTE.—Particulars of Appointments for insertion in this column will be welcomed.**

### Personalia.

CAPTAIN J. ST. R. WILSON, of Southport, who was assistant house surgeon at the Royal Hospital, Liverpool, when he engaged in military service, has been awarded the Military Cross.

THE following scholarships at St. Thomas' Hospital have been awarded: *Entrance Science Scholarships, 1st*, £150; *2nd*, £60.—J. O. Churcher and N. S. Macpherson, being equal, divide the two scholarships. *Arts Scholarships (two)*.—W. H. Dunn and E. G. Housden, each £15 15s.

THE Wellcome Historical Medical Museum will be closed for cleaning from September 1 to 30 inclusive.

### Manager's Notices.

**Now Ready, "The Hospital" Index to Vol. LXI., Oct. 1916 to April 1917. Price 2½d. per copy post free.**

**All letters on business matters must be addressed to The Manager, "The Hospital," 28 and 29 Southampton Street, London, W.C. 2.**

**Subscriptions**, which may commence at any time, are payable in advance. Cheques and Post-Office Orders should be crossed London County and Westminster Bank, Covent Garden Branch, and made payable to the Manager of THE HOSPITAL.

RATES OF SUBSCRIPTION (payable in advance).

| | | |
|---|---|---|
| UNITED KINGDOM. | | |
| Three Months (including Postage) | ... | 2s. 0d |
| Six Months do. | ... | 4s. 0d |
| Twelve Months do. | ... | 6s. 6d. |
| FOREIGN AND COLONIAL. | | |
| Three Months (including Postage) | ... | 2s. 6d. |
| Six Months do | ... | 5s. 0d. |
| Twelve Months do. | ... | 8s. 8d |

